# Coping strategies among adolescents and young adults living with HIV/AIDS in Accra-Ghana

**DOI:** 10.1186/s12889-023-17147-9

**Published:** 2023-11-27

**Authors:** Selom Dake, Harriet Affran Bonful, Vincent Ganu, Peter Puplampu, Alexander Asamoah, Hannah Ama Arthur, Linus Mwintuu, Emmanuel Asampong, Irene A. Kretchy, Adote Anum

**Affiliations:** 1 Department of Epidemiology and Disease Control, School of Public Health, University of Ghana, Accra, Ghana; 2 Department of Medicine, Korle Bu Teaching Hospital, Accra, Ghana; 3 Department of Medicine and Therapeutics, University of Ghana Medical School, Accra, Ghana; 4 Public Health Division, Ghana Health Service, Accra, Ghana; 5 Department of Social and Behavioural Sciences, School of Public Health, University of Ghana, Accra, Ghana; 6 Department of Pharmacy Practice and Clinical Pharmacy, School of Pharmacy, University of Ghana, Accra, Ghana; 7 Department of Psychology, University of Ghana, Accra, Ghana

**Keywords:** Coping strategies, Adolescents, Young adults, HIV/AIDS

## Abstract

**Background:**

Living with HIV/AIDS is remarkably stressful and has an adverse effect on one’s physical and mental health. In Sub-Saharan Africa, the introduction of highly active anti-retroviral therapy has led to an increased number of children with perinatal acquired HIV who are living into adolescence and adulthood. Developing strategies to cope with HIV becomes imperative, especially among these adolescents. The study determined the factors that influence coping strategies among adolescents living with HIV.

**Methods:**

An analytic cross-sectional design was used. A total of 154 adolescents aged 10–19 years living with HIV were systematically sampled at the Fevers Unit of Korle Bu Teaching Hospital from June to December, 2021. The adolescent version of the KidCope tool was used to assess the choice of coping strategies. Stata 16 was used to determine associations between independent variables and the coping strategies identified. Only variables that were significant at *p* = 0.1 or less in the crude model were used to run the adjusted regression model. The level of significance was set at *p* = 0.05 with a 95% confidence interval.

**Results:**

The mean age of participants was 19.2 ± 0.45 years with 51.9% (80/154) of participants being males. A majority, 57.1% of the participants employed positive coping strategies with 87.0% (135/154) using cognitive restructuring strategy. In an adjusted linear regression model, participants coping strategies were significantly associated with their educational level (*p* = 0.04) and presence of both parents as caregivers (*p* = 0.02).

**Conclusion:**

Participants largely adopted positive coping strategies in managing the disease. Factors that influenced the choice of coping strategies were higher levels of education and the presence of both parents as caregivers. The importance of a good social support structure and pursuing further education needs to be emphasized in counselling adolescents living with HIV as it promotes the choice of positive coping strategies.

## Background

HIV and AIDS is one of the world’s most devastating public health diseases of importance which has largely affected the global population. Globally, about 38 million people are estimated to be living with HIV and AIDS (PLHIV) with about 70% found in sub-Saharan Africa [1]. UNICEF fact sheet on HIV in 2019 further shows that the disease has affected all age groups. Adolescents and young adults between the ages of 10 and 24 living with HIV account for about 5% of the world’s HIV population with 1.5 million cases identified in Sub-Saharan Africa. In 2019, about 170,000 new cases were identified [2]. About 28,000 adolescents living with HIV die every year due to HIV-related complications with about 90% of these occurring in Sub-Saharan Africa. In 2021, about 27,500 HIV-associated mortalities occurred among adolescents globally and the 4th leading cause in African low- and middle-income countries [1].

Chronic illnesses like HIV/AIDS coupled with the energetic stage of life adolescents and young adults find themselves, makes it more difficult for them to cope with the stressors of life [3]. However, the advent of Highly Active Antiretroviral Therapy (HAART) and its successes, has helped these adolescents to have longer periods to live with the disease [4]. Multiple stressors about medication adherence, family financial problems, disease-related events, and disclosure have been identified in managing HIV/AIDS according to Anakwa and co-researchers in 2021 [5]. Poor coping strategies have many detrimental effects on the lives of the individual, the family structure, the community and nation [6]. They negatively affect the mental health of Adolescents Living with HIV (ALHIV), thereby exerting a toll on the human body. For example, the use of denial delays the onset of treatment and may cause progression of the disease with added complications that are difficult to manage [7]. It also affects the family as ALHIV push away their loved ones through their mental disengagement coping strategy leading to rifts and strife [8]. Poor coping strategies also lead to increased financial costs on the family as the ALHIV do not seek medical care early causing problems to fester and worsen [9]. The community and country are not spared as this promotes negative socioeconomic growth and increases the burden on the nation’s economic, health infrastructure, and social resources [9].

It is therefore necessary to develop positive coping strategies to reduce the psychological burden of ALHIV, resulting from all the difficulties related to this disease. Coping strategies consist of thoughts and behaviors that people use to consolidate the internal and external demands of stressful events and factors [10]. The potential of a stressor to cause mental health problems depends not only on the severity of the stress exposure but also on the resilience of the individual and the coping strategy employed [11]. Mental health experts have identified many stressors and their associated coping strategies among adolescents and older subgroups in managing HIV/AIDS.

Establishing and understanding the relationship between socio-demographic variables and coping strategy in the adolescent subgroup is key to improve clinical outcomes [12]. The application of this understanding by clinicians and other health professionals to manage chronic diseases as HIV and AIDS for various stressors related to a disease may favor the success of the entire therapeutic regimen.

There is however limited information on coping strategies and the factors that affect choice of coping strategies amongst adolescents living with HIV in Ghana. The coping strategies of these young adults living with HIV may be influenced by multiple factors including sociodemographic, individual factors, cultural and environmental factors, health status, religion, family characteristics, social networks, socioeconomic factors, relationship issues, etc. The objective of this study is to identify factors that affect the choice of coping strategies employed by adolescents and young adults living with HIV/AIDS.

## Method

A cross-sectional analytic study was carried out at the KorleBu Teaching Hospital (KBTH), one of the major hospitals located in Accra, the capital of Ghana. The study was carried out at the adolescent HIV clinic of the Hospital, from June to December 2021.

The Korle -Bu Teaching hospital is Ghana’s foremost referral facility located in the Accra Metropolis. Since it was established in 2003, it has been one of the many sites that provide care for persons living with HIV in Ghana.

All adolescents between the ages of 10–19 years as defined by WHO (26) who have been diagnosed as HIV positive and attend the HIV clinic at the Fevers Unit of the Korle Bu Teaching Hospital were included in the study.

All adolescents between the ages of 10–19 who were on admission at the Fevers Unit of the Korle Bu Teaching Hospital and those medically diagnosed with cognitive impairment as captured in their medical records were excluded. Recruitment of subjects was based on ALHIV who met the inclusion criteria.

The sample frame involved a list of all participants to be seen on their respective clinic days. The participants who attended the clinic on the respective clinic days usually Thursdays were offered cards with serial numbers grounded on a first come basis. The sampling interval used to systematically select the number of participants to be enrolled on a survey day at the clinic was determined by the sampling fraction, 20/60, where 60 is the total number of participants at the HIV clinic seen on a particular day and 20 the number of participants to be recruited on a clinic day. The first person was chosen randomly on survey day 1 and every third patient selected subsequently until the sample size was obtained.

A total sample size of 154 participants were recruited for the study using the formula for sample size calculations for a finite population.

### Data collection and analyses

Data were collected through interviews. The assessment was done in a sequential approach with participants being interviewed after they had finished with their clinical procedures.

The socio-demographic characteristic of participants such as age, sex, religious affiliation, level of education, occupation, parental status, and presence of caregivers were collected. Data were collected on common stressors the adolescents experienced daily using elements of a questionnaire employed by Folayan et al. to assess stressors [13]. The primary outcome, coping strategy, employed by adolescents living with HIV was measured using the adolescent version of the KidCope tool. The coping strategies were assessed using the Kidcope questionnaire used to measure cognitive and behavioural coping in adolescents [14]. The checklist asks respondents to rate the frequency and efficacy of coping strategies. These strategies include social support, problem-solving, social withdrawal, distraction, self criticism, cognitive restructuring, emotional expression, blaming others, resignation and wishful thinking [14]. The test–retest reliability of the Kidcope questionnaire was 0.41 to 0.83 over 3–7 days and 0.12 to 0.43 for 10 weeks. Items in the Kidcope questionnaire that were related to frequency are scored on a 4 point scale {not at all (0), sometimes (1), a lot (2) and almost all the time (3)} with a sum of the items giving a minimum total score of 0 and a maximum total score of 30. Items related to efficacy are rated on a 5-point scale {0- “Not at all” to 4- “very much”} [14]. For the purpose of this study, the focus was on the sum of the items related to frequency of coping strategy.

Data were entered into Microsoft excel 2016 and analyzed using STATA version 16.0 (StataCorp LLC). Tables were used to present the data on the socio-demographic variables, and the various stressors of the participants. For analysis of frequency of use of coping strategies, 6 of the items (distraction, social withdrawal, self criticism, blaming others, resignation and wishful thinking) were considered as negative coping strategies. The remaining 4 items (cognitive restructuring, problem solving, emotional expression and social support) were considered as positive coping strategies. Scores of each item considered as positive coping strategy were maintained as per the original scoring system. However all scores of each item considered as negative coping strategy were reversed (not at all (3), sometimes (2), a lot (1) and almost all the time (0)). After the reversal, scoring was then reclassified for all 10 items. For each question, any response with a score of 0 or 1 was assigned a score of *‘0’* and any response with a score of 2 or 3 was assigned a score of *‘1’*. For the new reclassification, each respondent will have a minimum score of 0 and a maximum score of 10. The mean score of the reclassified scores obtained for the study population was calculated. All persons with scores less than the mean were considered to have poor coping strategies and all those with scores equal to or above the mean were considered to have good coping strategies. The Chi-Square test was used to determine the association between the independent variables and the coping strategies identified. Fischer’s exact test was used where variables had less than five observations. A linear regression model was used to estimate the strength of association between the independent variables (social factors, demographic factors, and stressors) and the coping strategies. Only categorical variables with more than 20% of their observations having a value of five and below were significant at *p* = 0.1 or less were used to run the adjusted model. Where applicable, post estimation tests were performed to establish associations between categorical variables and the outcome variable in the adjusted model. The level of significance was set at a *p*-value of 0.05 with a 95% confidence interval. Shapiro Wilk ‘W’ test for normal data (*p* = 0.467) and Shapiro Francia ‘W’ test (*p* = 0.612) for normal data all showed that the scores were normally distributed. Tests for heteroskedasticity (Breusch-Pagan / Cook-Weisberg test for heteroskedasticity) were run and showed the assumption was not violated (chi2- 0.23, p-0.633). The Ramsey Reset Test using pairs of the fitted values of the score showed that the omitted variables assumption was not violated (F = 0.36, *p* = 0.78). The test of multicollinearity (Variance Inflation Factor Test) indicated that there was a lack of collinearity in the model (VIF < 2.5). The test of goodness of fit was included in the ordinary least square regression analysis model run to determine the linear relationship between the coping strategy and the independent variables (Adjusted R^2^- 0.11, F probability-0.0128).

## Results

### Socio-demographic characteristics of participants

The demographic characteristics of the participants are presented in Table 1. One hundred and fifty-four participants were recruited for this study. The mean age of participants was 19.2 years (95% CI: 18.7–19.6) with 51.9% of the participants being males. Most of the participants were students accounting for two-thirds of the study population. Although a significant proportion of the participants had both parents alive (61.1%), the caregiver for most of the participants was just a single parent (43.8%).

**Table 1:** Socio-demographic characteristics of ALHIV at the Fevers Unit, KBTH

Variable	Frequency (*N* = 154)	Percentage
**Age (years)**		
10–12	2	1.3
13–17	65	42.2
18–19	87	56.6
**Sex**		
Male	80	51.9
Female	74	48.1
**Religion**		
Christian	133	86.4
Muslim	21	13.6
Other	0	0
**Educational level**		
Primary	30	19.4
Secondary	97	62.9
Tertiary	23	14.9
Vocational/Technical	4	2.8
**Occupation**		
Student	101	69.5
Formally employed	25	16.2
Informally employed	8	5.2
Unemployed	14	9.1
Missing	6	
**Caregiver**		
Single Parent	67	43.8
Both Parents	53	34.6
Other	33	21.6
Missing	1	
**Parental Status**		
Both Parents Alive	99	61.0
One Parent Alive	54	36.4
No parents Alive	2	2.6
Total	154	100.0

Other-Grandparents, Uncles, Aunts, relatives of the extended family

### Stressors adolescents living with HIV face whilst living with the disease

Most of the participants admitted to dealing with stigma and discrimination (81.8%), taking drugs regularly (78.6%), and having to visit the hospital regularly (73.4%) as significant stressors in their lives. A summary of these stressors is presented in Table 2.

**Table 2:** Stressors of Adolescents Living with HIV at the Fevers Unit, KBTH

Stressors	Frequency (*N* = 154)	Percentage
**Having to take drugs regularly**		
Yes	121	78.6
No	33	21.4
**Body looking different from others**		
Yes	95	61.7
No	59	38.3
**Dealing with stigma and discrimination**		
Yes	126	81.8
No	28	18.2
**Thinking about death**		
Yes	43	27.9
No	111	72.1
**Having to visit the hospital regularly**		
Yes	113	73.4
No	41	26.6
**Telling my friends and family about my illness**		
Yes	95	61.7
No	59	38.3
**Having no friends**		
Yes	48	31.2
No	106	68.8
**People saying things about me that I don’t like**		
Yes	63	40.9
No	91	59.1
**Having arguments with my friends and family often**		
Yes	55	35.7
No	99	64.3
**Falling sick regularly**		
Yes	102	66.2
No	52	33.8

### Coping strategies employed by ALHIV

The coping strategies employed by the participants included cognitive restructuring (87.0%) and problem-solving (77.3%) as shown in Table 3.

**Table 3:** Coping strategies among ALHIV at the Fevers Unit, KBTH

Questions	Frequency (*N* = 154)	Percentage
**I tried to fix the problem by thinking of answers to it**		
Yes	119	77.3
No	35	22.7
**I blamed myself for the situation**		
Yes	55	35.7
No	99	64.3
**I usually blame someone else for causing the problem**		
Yes	50	32.5
No	104	67.5
**I tried to focus on the good side of the issue**		
Yes	135	87.0
No	19	13.0
**I just kept to myself**		
Yes	75	48.7
No	79	51.3
**I just wished the problem never happened**		
Yes	128	83.1
No	26	16.9
**I didn’t do anything because the problem couldn’t be fixed**		
Yes	85	55.3
No	69	44.7
**I tried to fix the issue by doing something/talking to someone**		
Yes	110	71.4
No	44	28.6
**I watched TV or playing a game to try and forget the problem**		
Yes	126	80.5
No	28	19.5
**I tried to calm myself down**		
Yes	124	80.4
No	30	19.6
**I tried to feel better by spending time with family and friends**		
Yes	108	70.1
No	46	29.9

### Association between socio-demographic characteristics and coping strategies of participants at the Fevers’ Unit KBTH

Results of the chi-square analyses showed significant associations between caregivers of the participants (Pearson chi^2^ = 7.36, *p* = 0.025) and positive coping strategies (Table 4).

**Table 4:** Association between socio-demographic characteristics and coping strategies of ALHIV at the Fevers, KBTH

	** Coping Strategy**			
**Characteristic**	**Positive**	**Negative**	**Chi** ^**2**^	***p-value***
**Gender**			2.96	0.085
Male	51(63.75)	29(36.25)		
Female	37(50.00)	37(50.00)		
**Religion**			0.23	0.635
Christian	77(57.89)	56(42.11)		
Muslim	11(52.38)	10(47.62)		
**Educational level**			4.43	0.218
Primary	19(63.33)	11(36.67)		
Secondary	50(82.47)	47(17.53)		
Tertiary	17(91.30)	6(9.70)		
Vocational/Technical	2(75.00)	2(25.00)		
**Occupation**			1.41	0.704
Student	63(58.88)	44(41.12)		
Formally employed	14(56.00)	11(44.00)		
Informally employed	5(62.50)	3(37.50)		
Unemployed	6(42.86)	8(57.14)		
**Parental Status**			2.02	0.364
Both Parents Alive	53(53.54)	46(46.46)		
One Parent Alive	34(62.96)	20(37.04)		
No Parents Alive	1(50.00)	1(50.00)		
**Caregiver**			7.36	**0.025†**
Single Parent	67(43.80)	30(44.77)		
Both Parents	53(34.60)	6(11.32)		
Other	33(21.60)	19(57.58)		

^**†**^ **-** Significant values at *p* < 0.05

### Association between stressors and coping strategies among ALHIV

No significant association was found between the stressors identified and coping strategies (Table 5).

**Table 5:** Association between stressors and coping strategies of ALHIV at the Fevers Unit, KBTH

		** Coping Strategy**			
**Characteristic**	**Total (%)**	**Positive**	**Negative**	**Chi** ^**2**^	***p-value***
**Having to take drugs regularly**				0.54	0.461
Yes	121	71(58.68)	50(41.32)		
No	33	17(51.52)	16(48.48)		
**Body looking different**				0.83	0.363
Yes	95	57(60.00)	38(40.00)		
No	59	31(52.54)	28(47.46)		
**Having no friends**				0.04	0.841
Yes	48	28(58.33)	20(41.67)		
No	106	60(56.60)	46(43.40)		
**Dealing with stigma**				1.61	0.205
Yes	126	69(54.76)	57(45.24)		
No	28	19(67.86)	9(32.14)		
**Thinking about death**				0.87	0.351
Yes	43	22(51.16)	21(48.84)		
No	111	66(59.46)	45(40.54)		
**Having to visit hospital reg**				1.73	0.188
Yes	113	61(53.98)	52(46.02)		
No	41	27(57.14)	14(34.15)		
**Telling friends/family about my illness**				0.18	0.667
Yes	95	53(55.79)	42(44.21)		
No	59	35(59.32)	24(42.86)		
**Having arguments with family/friends**				0.28	0.593
Yes	55	33(60.00)	22(40.00)		
No	99	55(55.56)	44(44.44)		
**Falling sick regularly**				2.18	0.140
Yes	102	54(52.94)	48(47.06)		
No	52	34(65.38)	18(34.62)		

Table 6 summarizes the results of the crude and adjusted multivariable linear regression analysis of the association between coping strategy, stressors, and sociodemographic factors. Significant differences in coping strategy index scores were observed across secondary levels of education (*p* = 0.012) and the presence of both parents as caregivers (*p* = 0.006) after postestimation tests to establish associations between categorical variables and coping strategies in the adjusted model.

**Table 6:** Crude and adjusted linear regression analysis of the association between Coping Strategies (composite scores) and characteristics of ALHIV at the Fevers Unit, KBTH

	**Crude linear regression**				**Adjusted linear regression**			
		**95% CI**				**95% CI**		
**Characteristic**	**Coefficient (B)**	**Lower**	**Upper**	***p-value***	**Coefficient (B)**	**Lower**	**Upper**	***p-value***
**Educational level**								* **0.041**
Primary	Ref				Ref			
Secondary	-0.95	-1.77	-0.14	0.022	-0.94	-1.68	-0.20	0.012
Tertiary	-1.56	-3.69	0.56	0.148	-1.59	-3.47	0.28	0.096
Vocational/Technical	-0.66	-1.85	0.52	0.273	-0.34	-1.31	0.64	0.491
**Parental Status**								
Both Parents Alive	Ref				Ref			0.205
One Parent Alive	0.70	-0.09	1.50	0.083	0.65	-0.06	1.37	0.076
No Parents Alive	-0.18	-4.06	3.71	0.929	0.21	-3.34	3.74	0.909
**Caregiver**								* **0.021**
Single Parent	Ref				Ref			
Both Parents	-1.15	-1.98	-0.32	0.007	-1.09	-1.85	-0.32	0.006
Other	-0.24	-1.05	0.56	0.548	-0.24	-0.97	0.49	0.519

^*^P-values obtained from post-estimation tests

## Discussion

The main objective of the study was to assess the coping strategies among adolescents living with HIV at the adolescent HIV clinic of the Fevers Unit, KBTH. The most used coping strategy was cognitive restructuring with about 87% of participants employing that to cope with living with the disease. This is comparable with a study conducted by Folayan et al., (2017) which identified cognitive restructuring as the most common coping mechanism used to manage stressors associated with the disease [8].

Emotional regulation was the least used coping strategy among the participants (32.5%). This is in stark contrast to studies conducted in the USA which reported emotional regulation as the most frequently used coping mechanism [15]. This contrast may be due to several factors. Comparisons between high- and low-income countries show a significant difference in the presence of a mental health workforce of psychiatrists, nurses, psychologists, and social workers [16]. Individuals trained to impart skill in emotional regulation are thus fewer leading to its rather sparse use in the population. Also, cultural differences in the approach to problems faced in everyday life may account for the differences in managing stressors [17]. The premium placed on managing emotions in Lower Middle-Income Countries (LMIC) may be different from those in high-income countries.

Generally, a majority of participants (57.1%) used positive coping strategies in managing the challenges they encounter whilst living with HIV. This is in keeping with many other studies conducted in the USA, Nigeria, and Tanzania which serve to highlight the effort that has been made to raise awareness on the importance of mental health in dealing with living with chronic diseases [8, 15, 18].

Of all the maladaptive strategies employed, wishful thinking recorded the highest frequency with about 83.1% of participants using this method. This is comparable with studies in India which identified blaming others and wishful thinking as common tools used by participants living with HIV in managing the disease [19]. Negative coping strategies have been associated with poor mental health and adverse clinical outcomes in adolescents living with HIV. It is therefore important that the use of positive coping strategies be encouraged as this greatly helps to reduce the morbidity and mortality associated with living with HIV [20].

Most of the participants admitted to dealing with stigma and discrimination (81.8%), antiretroviral medications on a daily basis (78.6%), and having to visit the hospital regularly (73.4%) as stressors in their lives among others. Our findings are comparable to findings in other contexts. For example, in Uganda, researchers identified that dealing with stigma is the most common stressor adolescents living with HIV face [21]. Disclosure (61.7%), adherence to medication (78.6%), and stressors associated with the management of disease-related states such as falling sick often (66.2%) and visiting the hospital regularly (73.4%) were also identified. All these are consistent with current literature that identified these factors as stressors in the lives of ALHIV [8, 21, 22].

### Factors associated with coping strategies

#### Demographic characteristics of ALHIV

Higher levels of education significantly increased coping strategy index by 0.94 when comparing adolescents living with HIV who had secondary education to adolescents living with HIV who had primary education. This is also not an isolated finding. Similar findings that showed that higher levels of education led to better coping strategies, better clinical outcomes and better quality of life have been reported in other contexts [8, 23].

#### Social support

Social support also plays a role in how adolescents cope with the stressors HIV places on them [24]. Of important note is the impact of the presence of both parents as caregivers. This led to a 1.15 increase in coping strategy index as compared to a single parent as caregiver. This is comparable to studies among HIV affected families in China that highlighted the benefits of both parents being present as caregivers in managing the difficulties HIV AIDS poses [25].

Improved ability to cope has been shown to lead to improvement in mental health and the ability to deal with the stress living with HIV poses. Social support is an important modulator in this. Parents provide emotional support and help educate their adolescents on how to deal with stressors that they may face. Daily hassles, poor coping and limited social support can adversely affect the psychological well-being of HIV affected adolescents [22]. Studies among HIV affected adolescents in Zambia and Nigeria help to buttress this important point [8, 18].

The factors that affect the coping strategies need to be utilized positively by the ALHIV, the clinicians who manage them and the organizations in charge of policies that affects these adolescents. This will help to improve their mental health and thus their general wellbeing. The Ghana AIDS Commission together with the Ministry of Health need to target these areas in order to be able to affect how these adolescents manage living with the disease.

### Study limitation

Only one hospital was used for the study and may not necessarily reflect the experiences of other adolescents and young adults living with HIV in the country. Generalizability of the results to the rest of the population may be difficult. Also, the study is cross sectional and therefore difficult to assess whether poor coping strategies existed before changes in some of the independent variables occurred. Furthermore, responses to patient’s choice of coping were largely based on recall and thus may be subject to recall bias.

## Conclusion

The study shows that adolescents living with HIV largely employ positive coping strategies in managing stressors associated with living with HIV. Cognitive restructuring was the most used coping strategy. Socio-demographic factors significantly associated with a higher coping index score included attaining a higher level of education. The presence of a social support structure significantly predicted choosing more beneficial coping strategies as it led to a higher coping strategy index. This was made more apparent when both parents were active as caregivers in the lives of young adults living with HIV. In order to promote the use of positive coping strategies, counselling sessions held for ALHIV should stress on the importance of a good social support structure and pursuit of further education.

## Data Availability

Data are available from the corresponding author (habonful@ug.edu.gh) upon reasonable request.

## Abbreviations

ALHIV: Adolescents Living with HIV
HAART: Highly Active Antiretroviral Therapy
LMIC: Lower Middle-Income Country
KBTH: Korle Bu Teaching Hospital
